# Intestinal Fibrosis and Gut Microbiota: Clues From Other Organs

**DOI:** 10.3389/fmicb.2021.694967

**Published:** 2021-07-16

**Authors:** Shukai Zhan, Na Li, Caiguang Liu, Ren Mao, Dongxuan Wu, Tong Li, Minhu Chen, Xiaojun Zhuang, Zhirong Zeng

**Affiliations:** Department of Gastroenterology, The First Affiliated Hospital, Sun Yat-sen University, Guangzhou, China

**Keywords:** intestinal fibrosis, fibrogenesis, gut microbiota, microbiota alteration, metabolites

## Abstract

Fibrosis is a complex and difficult to elucidate pathological process with no available therapies. Growing evidence implicates intestinal microbiota in the occurrence and development of fibrosis, and the potential mechanisms involved in different organs have been explored in several studies. In this review, we summarize the causative and preventive effects of gut microbiota on intestinal fibrosis, as well as the relationships between gut microbiota and fibrosis in other organs. Interestingly, several colonized microbes are associated with fibrosis via their structural components and metabolic products. They may also play essential roles in regulating inflammation and fibroblast activation or differentiation, which modulates extracellular matrix formation. While the relationships between intestinal fibrosis and gut microbiota remain unclear, lessons can be drawn from the effects of gut microbiota on hepatic, cardiac, nephritic, and pulmonary fibrosis. Various intestinal microbes alterations have been detected in different fibrotic organs; however, the results were heterogeneous. Mechanisms by which the intestinal microbiota regulate fibrotic processes in other organs, such as novel metabolic products or specific microbes, are also discussed. The specific microbiota associated with fibrosis in other organs could instruct future studies aiming to discover prospective mechanisms regulating intestinal fibrosis.

## Introduction

Fibrosis is a widespread pathological process that affects almost every organ and has poor therapeutic efficacy. It gives rise to end-stage organ failure and aggravates dysfunction, and is a leading cause of mortality worldwide (Liu, 2011; Tsochatzis et al., 2014; Bettenworth and Rieder, 2017; Richeldi et al., 2017; Rosenbloom et al., 2017; Di Ciaula et al., 2020). Fibrosis is the pathological overabundance of extracellular matrix (ECM), which contains several molecular components, including collagens, glycoproteins, and proteoglycans. Physiologically, the ECM maintains a homeostatic balance of synthesis and degradation through complicated regulatory pathways, facilitating wound healing and tissue recovery after injury or inflammation under healthy conditions (Speca et al., 2012; Friedman et al., 2013). However, overstimulation by excessive inflammation or pathologic environmental factors activates mesenchymal cells such as myofibroblasts, smooth muscle cells to continuously proliferate. These cells are characterized by ECM secretion and their abnormal increase will disturb leads to fibrosis (Stallmach et al., 1992; Wynn, 2008). In addition, non-mesenchymal cells, such as fibrocytes, endothelial cells and epithelial cells can transform into fibroblasts when stimulated. This also contributes to ECM accumulation (Kalluri, 2009; Rieder, 2013). The innate and adaptive immune reactions play central roles in bridging causative factors and their effects on fibrosis. The immune system is a highly complex network of various cell types, in which molecules such as cytokines, chemokines, growth factors, angiogenic factors, and reactive oxygen species (ROS), play roles in intercellular communication. Immune system activation by heterogeneous stimuli can activate mesenchymal and non-mesenchymal cells either directly or indirectly, by modulating inflammation (Speca et al., 2012; Rieder, 2013; Tsochatzis et al., 2014).

The gastrointestinal tract is heavily colonized by diverse microbes, including bacteria, fungi, viruses, and parasites (Tremaroli and Bäckhed, 2012; Kamada et al., 2013). Bacteria comprise the majority, up to 100 trillion microbial cells and 1,000 different species (Qin et al., 2010). Recently developed high-throughput sequencing technology and data analysis methods have illustrated the gut microbiome much more precisely than traditional culture-based techniques. This has enabled the study of specific species and even certain microbial structures (Liu et al., 2020). The composition and quantity of this microbial community is relatively homeostatic, depending on various factors such as the dietary patterns, living environment, and health of the host (Dominguez-Bello et al., 2011; Marques et al., 2017). Dysbiosis, which involves compositional alterations in gut microbiota, can increase the risk of disease (Tremaroli and Bäckhed, 2012; Bajaj, 2019).

Gut microbiota, as important environmental factors, are attracting increasing attention in the development of fibrogenesis (Lau et al., 2017; Acharya and Bajaj, 2019; Plata et al., 2019). To date, there have been many studies regarding gut microbial effects on hepatic, cardiac, and nephritic fibrosis; in contrast, the impact on intestinal fibrosis has been only studied sporadically (Speca et al., 2012; Minicis et al., 2014; Yiu et al., 2014; Karbach et al., 2016; Dornas and Lagente, 2019). Unlike other organs, the gastrointestinal tract has a direct and close connection to gut microbiota; therefore, the microbial effects and mechanisms underlying intestinal fibrosis may be unique, and warrant further exploration. Here, we review the latest studies and integrate their findings to summarize the known microbial influences on intestinal fibrosis. Information regarding the effects of intestinal microbiota on fibrosis in other organs is also included.

## Gut Microbiota and Intestinal Fibrosis

Intestinal fibrosis occurs in many gastrointestinal tract diseases, including inflammatory bowel disease (IBD), solitary rectal ulcers, radiation enteropathy, and eosinophilic enteropathy, and results in intestinal stenosis and obstruction. Almost all studies investigating relationships between intestinal fibrosis and gut microbiota have focused on IBD (Van Assche et al., 2004; Burke et al., 2007; Wynn, 2008). Encompassing both Crohn’s disease (CD) and ulcerative colitis, IBD is a lifelong relapsing and remitting inflammatory condition of the gastrointestinal tract, which is mainly associated with the activation of intestinal fibroblasts to increases collagen synthesis and facilitate fibrosis (Stallmach et al., 1992). Intestinal fibrosis is detected most frequently in patients with IBD, especially those with CD (Van Assche et al., 2004). Despite the emergence of effective biological therapies for intestinal inflammation, no effective fibrosis treatments exist currently. Fibrosis occurs in more than one-third of patients with CD, causing intestinal obstructions that require surgery in 30–50% of patients within 10 years of disease onset (Cosnes et al., 2002; Pariente et al., 2011). Treatments, such as surgical resection, endoscopic dilation, or section, are all temporary measures, and recurrence rates increase with time (Burke et al., 2007; Rieder et al., 2017).

The close relationship between intestinal microbiota and fibrosis was identified decades ago. After inoculating a fecal suspension sourced from healthy specific pathogen free (SPF) rats into the colonic walls of germ-free mice, researchers noticed obvious increases in collagen accumulation in inoculated regions (Mourelle et al., 1998). Moreover, some patients with CD have circulating antibodies against microbial antigens from *Saccharomyces cerevisiae* or *Pseudomonas fluorescens*, which are correlated with the clinical characteristics of intestinal fibrotic stenosis as well as surgical interventions (Mow et al., 2004). These results indicate that gut microbiota can induce fibrosis both directly and indirectly.

All intestinal immune and non-immune cells express pattern recognition receptors (PRRs), such as extracellular toll-like receptors (TLRs) and intracellular nucleotide oligomerization domain-like receptors (NLRs), which recognize pathogen-associated molecular patterns (PAMPs) and transmit intracellular signals. PAMPs are microbe-derived molecules, including components of bacterial cell walls, DNA, and double-stranded RNA (dsRNA) (Liew et al., 2005). When intestinal epithelial barrier integrity is disrupted by dysbiosis and inflammation, gut microbes are continuously exposed to immune or mesenchymal cells and activate intracellular signaling through their corresponding TLRs.

**FIGURE 1:**
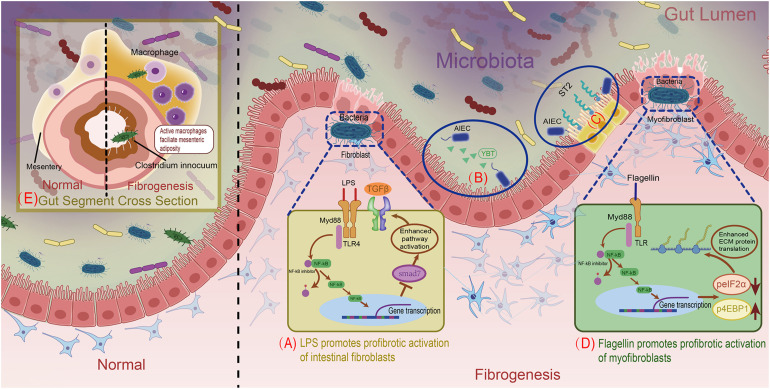
Overview of mechanisms for gut microbiota facilitating intestinal fibrosis. **(A)** LPS promotes profibrotic activation of intestinal fibroblasts. **(B)** AIEC’s siderophore Ybt strengthens its intestinal subepithelial localization. **(C)** AIEC’s flagellin stimulates intestinal epithelial cells to express the IL33 receptor. **(D)** Bacterial flagellin increases ECM proteins through modulating in the post-transcriptional level. **(E)** *C. innocuum’s* translocation to mesenteric adipose drives macrophages activation, resulting in fibrogenesis.

Specially, lipopolysaccharide (LPS), a component of the outer membranes of Gram-negative bacteria, is a type of PAMP proven to be fibrogenic. When fibroblasts are exposed to LPS, as mentioned previously herein, this molecule is recognized by corresponding TLR4 in the fibroblast membrane. After that, TLR4 oligomerizes and recruits downstream adaptors to its cytoplasmic toll-interleukin-1 receptor (TIR) domains. The following signaling event can be separated into two pathways, which are dependent and independent of the specific TIR domain-containing adaptor protein, myeloid differentiation primary response gene 88 (MyD88), respectively. The MyD88-dependent pathway leads to phosphorylation and degradation of inhibitory nuclear factor-κB (NF-κB) members, resulting in translocating NF-κB to nucleus and regulating gene transcription (Dauphinee and Karsan, 2006; Lu et al., 2008). Though the underlying mechanism hasn’t been unveiled, it’s known that gene transcription regulation will suppress the expression of SMAD family member 7 (SMAD7), a negative regulator of transforming growth factor β1 (TGFβ1) signaling, leading to enhanced TGFβ1 signaling and increased ECM protein secretion (see Figure 1A; Burke et al., 2010; Frangogiannis, 2020). Furthermore, when human fibrocytes were exposed to LPS, they produced higher amounts of collagen than when exposed to TGFβ1, indicating that LPS can enhance fibrosis independently of inflammatory TGFβ1 stimulation (Sazuka et al., 2014). Another bacterial cell wall polymer, peptidoglycan–polysaccharide, may also increase TGFβ1 expression and collagen accumulation in myofibroblasts through similar preceding mechanisms (van Tol et al., 1999).

Contrary to traditional research which studied the composition of the gut microbiome as a whole, recent studies use various models to explore the roles of specific microbes in fibrosis. *Mucispirillum schaedleri* and *Ruminococcus* in the cecum as well as *Streptococcus* and *Lactobacillus* in the ileum were positively correlated with fibrosis in a murine model of transgenic tumor necrosis factor-like cytokine 1A (TL1A) overproduction (Jacob et al., 2018). TL1A is a member of the tumor necrosis factor superfamily that is expressed in many cell types, including immune cells, epithelial cells, and fibroblasts, in response to stimulation by TLR or other molecules such as interleukin (IL)1. Its overexpression in mice leads to spontaneous enteritis and fibrosis (Zheng et al., 2013). Interestingly, gut microbiota are essential for this pathologic process, as they augment fibroblast adhesion, migration, and differentiation into myofibroblasts. However, *Oscillospira*, *Coprococcus*, *Faecalibacterium prausnitzii*, and *Bacteroides* were negatively correlated with fibrosis (Jacob et al., 2018).

Similarly, *Ruminococcus* were implicated in stricture development in a multicentric, prospective inception cohort study composed of pediatric patients with newly diagnosed CD (Kugathasan et al., 2017). However, another study discovered that *Lactobacillus acidophilus* improved inflammation-dependent fibrosis in a dextran sodium sulfate (DSS)-induced chemical enteritis model (Park et al., 2018). The exact mechanisms through which these microbes affect fibrogenesis are unknown.

Adherent/invasive *Escherichia coli* (AIEC) is an intestinal *E. coli* subset that is enriched in patients with CD. As AIEC is most frequently isolated from the terminal ileum, which is also the most common site of fibrotic stricture in CD, it is conceivable that AIEC contributes to fibrosis (Rieder et al., 2017; Darfeuille-Michaud et al., 2004). Persistent AIEC infection can augment intestinal inflammation, leading to inflammation-dependent fibrosis (Small et al., 2013). Specifically, the siderophore yersiniabactin (Ybt) of AIEC, which originally sequesters and imports extracellular metals, plays a non-canonical role in fibrosis development (Perry and Fetherston, 2011). Upon its release from AIEC, Ybt strengthens bacterial subepithelial localization to facilitate inflammation-associated fibrogenesis through colonization in the inflammation-prone *Il10*−/− mouse model (see Figure 1B; Kim et al., 2005; Ellermann et al., 2019). In addition, AIEC flagellin was studied in DSS-induced mice and modified *Salmonella enterica* serovar Typhimurium-infected mice, in which CD-like colitis was persistent while fibrosis was absent. After AIEC colonization, flagellin stimulation of intestinal epithelial cells significantly increased expression of the IL33 receptor growth stimulation expressed gene 2 (ST2), resulting in the activation of profibrotic NFκB and IL13 (see Figure 1C; Schmitz et al., 2005; Fichtner-Feigl et al., 2007; Imai et al., 2019). In an *in vitro* intestinal myofibroblast model, bacterial flagellin reduced phosphorylation of eukaryotic initiation factor 2α (eIF2α) and increased phosphorylation of eukaryotic translation initiation factor 4E (eIF4E)-binding protein 1 (4EBP1) via the MyD88-dependent TLR pathway. Phosphorylation of eIF2α blocked pre-initiation mRNA translation complex formation and inhibited translation, while 4EBP1 activation was enhanced (Kimball, 1999; Grewal, 2009), facilitating the translation and accumulation of intracellular ECM proteins (see Figure 1D; Zhao et al., 2020).

Recently, *Clostridium innocuum* was discovered to translocate from the gut lumen to the mesenteric adipose tissue (MAT) of surgically resected CD samples. This was confirmed using gnotobiotic mice raised with altered Schaedler flora (ASF), a well-defined consortium of eight anaerobic bacterial species (*L. acidophilus*, *Lactobacillus murinus*, *Bacteroides distasonis*, *M. schaedleri*, *Eubacterium plexicaudatum*, and two *Clostridium* species) that promote healthy gut development. After DSS-treated ASF mice were irrigated with *C. innocuum*, the bacteria were found in the MAT and selectively activated specific profibrotic macrophages in chronically inflamed states. Active macrophages elicited various cytokines or growth factors, leading to mesenteric adiposity and fibrosis. This process is thought to protect the host from the translocation of other microbes or bacterial products into the circulation (see Figure 1E; Schaedler et al., 1965; Ha et al., 2020). Apart from bacteria, which predominate the intestinal microbiome, the genus *Anaeroplasma* is also positively associated with intestinal fibrosis (Burke et al., 2010).

## Gut Microbiota and Hepatic Fibrosis

Hepatic fibrosis, or cirrhosis, is mainly caused by chronic liver damage due to viral infection, alcohol abuse, or non-alcoholic liver disease (Tsochatzis et al., 2014). The liver synthesizes and secretes bile acids and other mediators into the gut lumen through the bile system, contributing to normal intestinal microbiota homeostasis and metabolism (Ridlon et al., 2015). Conversely, homeostatic gut microbiota compose an integral intestinal barrier; as a result, few of their components and metabolites enter the liver through the portal vein, and are cleared by hepatic mononuclear phagocytes (Tripathi et al., 2018).

In chronic liver disease, the intestinal barrier is impaired, leading to dysbiosis or bacteria overgrowth in the small intestine. Alterations in intestinal microbes play a reactive role in increasing intestinal permeability (Wigg et al., 2001). A leaky gut allows many more microbes and microbial components to enter the liver through circulation. Bacteria, LPS, endotoxins, and even bacterial DNA have been found in the blood of patients and various animal models of chronic liver disease (Volynets et al., 2012; Yan and Schnabl, 2012; Hawkesworth et al., 2013; Zhou and Zhong, 2017). These PAMPs are recognized by PRRs on hepatocytes, Kupffer cells, hepatic stellate cells (HSCs), and endothelial cells. Along with the aforementioned LPS–TLR4 signaling axis, hepatic dsRNA-TLR3, flagellin-TLR5, and RNA-NLR1 axes have also been identified (Jäckel et al., 2017; Chen et al., 2019). When HSCs were stimulated directly through PRRs or indirectly via inflammation, ECM synthesis, and deposition increased (Elpek, 2014).

As most studies regarding the relationships between liver fibrogenesis and gut microbiota have focused on PAMPs, the roles of specific microbes in hepatic fibrosis remain mysterious. Moghadamrad et al. (2019) treated ASF-colonized mice and SPF mice with carbon tetrachloride (CCl_4_) to induce cirrhosis, and discovered that the ASF microbes promoted CCl_4_-induced liver fibrosis, indicating that gut dysbiosis contributes to cirrhosis through an undefined mechanism.

Several studies have examined gut microbiota changes in cirrhosis. Intestinal dysbiosis varies in cirrhosis, depending on its etiology, the disease period, and the patient’s diet (Bajaj et al., 2018). For example, patients with non-alcoholic steatohepatitis (NASH) without fibrosis exhibited a predominance of *Bacteroides*, while those with fibrosis had higher relative abundances of *Ruminococcus* other than *Bacteroides* (Boursier et al., 2016). Compared with healthy controls, patients with alcoholic cirrhosis had decreased *Bacteroides*, *Parabacteroides*, *Prevotella*, and *Clostridium*, and increased *Lactobacillus* and *Bifidobacteria* (Dubinkina et al., 2017). In another study on cirrhosis, *Streptococcus* and *Veillonella* increased while *Bacteroidetes* and *Eubacterium* decreased (Qin et al., 2014). A study of patients with viral and alcohol-related cirrhosis revealed reduced *Bacteroidetes* and increased *Proteobacteria* and *Fusobacteria*. More specifically, *Enterobacteriaceae*, *Veillonellaceae*, and *Staphylococci* were abundant (Chen et al., 2011). In patients with alcoholic cirrhosis, *Enterobacteriaceae* and *Halomonadaceae* were increased while *Lachnospiraceae*, *Ruminococcaceae*, and *Clostridiales XIV* were decreased compared to abundances in those with non-alcoholic cirrhosis. However, in patients with NASH cirrhosis, the families *Bacteroidaceae* and *Porphyromonadaceae* from the phylum *Bacteroidetes* were enriched and *Veillonellaceae* was decreased compared to abundances associated with cirrhosis of other etiologies (Bajaj et al., 2014).

Furthermore, intestinal microbiota components differ between compensated and decompensated cirrhosis (Bajaj et al., 2018). *Staphylococcae* and *Lachnospiraceae* levels are positively and negatively correlated with the Child–Pugh score, respectively. The microbiota also vary in cirrhosis with and without complications, such as hepatic cell carcinoma, hepatic encephalopathy, and infection (Bajaj et al., 2014; Grąt et al., 2016).

## Gut Microbiota and Cardiac Fibrosis

Intestinal microbiota likely fail to directly contact cardiac tissue because of the intestinal barrier, hepatic clearance, and the heterogeneous immune cells in the bloodstream. Therefore, they primarily induce cardiac fibrosis through various molecules, including structural components and metabolites. Cardiac fibrosis results in cardiac insufficiency, which inhibits intestinal venous return, causing overgrowth of anaerobic bacteria and increasing intestinal permeability (Sandek et al., 2007, 2014; Schiattarella et al., 2017).

Several studies have identified intestinal microbiome changes in cardiac insufficiency. One found abnormally high levels of the *Proteobacteria* genera *Campylobacter*, *Shigella*, and *Salmonella* as well as the *Firmicutes* genus *Lactobacillus* in patients with chronic heart failure (Pasini et al., 2016; Kamo et al., 2017). In another study, patients with stable cardiac insufficiency had smaller proportions of *Lachnospiraceae* compared to controls (Kummen et al., 2018). Decreases in the families *Coriobacteriaceae*, *Erysipelotrichaceae*, and *Ruminococcaceae* and the genera *Blautia*, *Collinsella*, uncl. *Erysipelotrichaceae*, and uncl. *Ruminococcaceae* were found in patients with heart failure compared to controls (Luedde et al., 2017).

Gut microbiota facilitate cardiac fibrosis, and this was straightly proven with the use of germ-free animal models, though the specific effective factor was not explored at that time (Karbach et al., 2016). Currently, bacterial metabolic products are thought to be contributive. Of these, trimethylamine N-oxide (TMAO) is the most important and well-studied. Dietary phosphatidylcholine, choline, and carnitine are metabolized by intestinal microbiota in the lumen, producing trimethylamine (TMA). TMA is absorbed and travels through the portal vein to the liver, where it is oxidized before entering systemic circulation (Tang et al., 2019). In cardiac fibroblasts, TMAO enhances TGFβ receptor I expression and inhibits the expression of SMAD2, a downstream inhibitor of TGFβ signaling. By facilitating TGFβ signaling, TMAO promotes cardiac fibroblast differentiation into myofibroblasts, causing cytokine secretion, and cardiac fibrosis. This was confirmed in a high choline diet mouse model (Yang et al., 2019). LPS can also aggravate cardiac fibrosis via LPS–TLR4 signaling (Frangogiannis, 2014).

In another mouse model of heart failure, researchers found that a high-fiber diet increased the proportion of *Bacteroides acidifaciens* and ameliorated cardiac fibrosis. Although the underlying mechanism is unclear, decreased synthesis of a master cardiovascular regulator, early growth response protein 1, was suggested (Marques et al., 2017).

## Intestinal Microbiota and Nephritic Fibrosis

Renal fibrosis is a pathological process involved in different types of chronic kidney disease (CKD) that leads to loss of renal function (i.e., end-stage renal disease). Intestinal dysbiosis occurs during CKD, and microbiome changes result in aberrant metabolism and increases in pernicious by-products, most of which are uremic toxins. Similar to that in cardiac disease, the enhanced intestinal permeability in CKD results in increased uremic toxins in the systemic circulation, aggravating nephritic fibrosis (Meijers et al., 2018).

TMAO also causes nephritic fibrosis. It mainly acts on SMAD3, another signaling molecule downstream of TGFβ. When mice were fed a diet supplemented with TMAO or high fat, enhanced SMAD3 phosphorylation, tubulointerstitial fibrosis, and collagen deposition were observed. These results were verified in a CKD cohort (Tang et al., 2015; Sun et al., 2017; El-Deeb et al., 2019).

*p*-Cresyl sulfate is a uremic toxin produced by aberrant intestinal microbes in nephritic fibrosis. Further, it is a sulfated conjugate of hepatic p-cresol. The increased anaerobic bacteria in the guts of patients with CKD metabolize the dietary amino acids tyrosine and phenylalanine to synthesize p-cresol, which travels though anion transporters and accumulates intracellularly, leading to ROS generation through nicotinamide adenine dinucleotide phosphate oxidase activation. Enhanced oxidative stress activates TGFβ signaling, resulting in collagen accumulation (Miyamoto et al., 2011; Watanabe et al., 2013).

Moreover, a series of tryptophan metabolites, including indole acetic acid, indole lactic acid, and tryptamine, are generated by almost all intestinal microbes. Diverse metabolic products are produced by multiple microbes, forming a complicated network involving heterogeneous pathways that control renal fibrosis by adjusting gene expression. These metabolites were recently reviewed by Liu et al. (2021). In addition, decreased microbial production of short chain fatty acids (SCFAs) like valproic acid or butyrate may contribute to nephritic fibrosis. SCFAs are general histone deacetylase inhibitors that suppress TGFβ signaling, preventing pericyte differentiation into myofibroblasts (Matsumoto et al., 2006; Zhang et al., 2018).

While characterizing the intestinal dysbiosis in nephritic fibrosis, one study comprising 24 stable patients with end-stage renal disease found increases in the bacteria families *Alteromonadaceae*, *Cellulomonadaceae*, *Clostridiaceae*, *Dermabacteraceae*, *Enterobacteriaceae*, *Halomonadaceae*, *Methylococcaceae*, *Micrococcaceae*, *Moraxellaceae*, *Polyangiaceae*, *Pseudomonadaceae*, *Xanthomonadaceae*, and *Verrucomicrobiaceae*, and decreases in *Lactobacillaceae* and *Prevotellaceae* (Wong et al., 2014).

## Intestinal Microbiota and Pulmonary Fibrosis

A multitude of causative factors for pulmonary fibrosis have been identified, including autoimmune disorders, silica inhalation, and radiotherapy (Moore and Moore, 2015). However, like in other organs, the relationships between pulmonary fibrosis and gut microbiota are relatively unexplored, and most studies have focused on the roles of airway microbiota (Takahashi et al., 2018; Zhang et al., 2020). Recently, two studies investigated gut microbiota variations in patients and animal models of pulmonary fibrosis. In patients with silica-induced pulmonary fibrosis, species of the genera *Megamonas* and of the phyla *Proteobacteria*, *Synergistetes*, *Lentisphaerae*, *Tenericutes*, and *Cyanobacteria* increased, while *Pseudomonas aeruginosa*, species of the genera *Subdoligranulum*, *Blautia*, *Nitrosomonadaceae*, and *Bifidobacterium*, and the phyla *Actinobacteria*, *Acidobacteria*, *Gemmatimonadetes*, *Saccharibacteria*, *Fusobacteria*, *Aminicenantes*, and *Verrucomicrobia* decreased (Zhou et al., 2019).

## Discussion

Changes in intestinal microbiota and their products will activate various immune and non-immune cells, causing inflammation and stimulating mesenchymal cells to produce ECM. This common mechanism underlies almost all fibrotic processes. Nevertheless, its specific presentation varies dramatically in different organs. In the gut and liver, which are directly exposed to the intestinal microbiota, certain bacteria, or their components have been identified as fibrotic promotors, while in the lung, heart, and kidney, the effects of intestinal microbiota involve their by-products, making it difficult to identify suspected microbes. Multiple microbial products strongly induce fibrosis in these organs (see Table 1).

**TABLE 1:** Overview of intestinal microbial impacts on fibrogenesis in different organs.

**Bacterial species/structure/metabolites**	**Impacts on organs**	**Impacts on fibrogenesis**	**Pathways**	**Models**	**References**
**Gut bacterial species**					
*Mucispirillum schaedleri*, *Ruminococcus*, *Streptococcus*, *Lactobacillus genera*	Intestine	Facilitated	Intestinal fibroblast activation and differentiation↑	TL1A overexpression mice	Jacob et al., 2018
AIEC	Intestine	Facilitated	Ybt releasing/AIEC subepithelial localization↑/TGF-β1↑	IL10−/− mice	Ellermann et al., 2019
AIEC	Intestine	Facilitated	Flagellin/intestinal epithelial cells express ST2↑/IL-33 signal (NF-κB, IL-13)↑	Modified *S. typhimurium*-infected mice, DSS-treated mice, intestinal epithelial cells	Imai et al., 2019
*Clostridium innocuum*	Intestine	Facilitated	Translocation to MAT/macrophages activation	ASF mice	Ha et al., 2020
*Ruminococcus*	Intestine	Facilitated	N/A	Human	Kugathasan et al., 2017
*Oscillospira, Coprococcus*, *Faecalibacterium prausnitzii*, *Bacteroides*	Intestine	Attenuated	N/A	TL1A overexpression mice	Jacob et al., 2018
*Lactobacillus acidophilus*	Intestine	Attenuated	N/A	DSS-treated mice	Park et al., 2018
ASF	Liver	Facilitated	N/A	CCl4-treated mice	Moghadamrad et al., 2019
*Bacteroides acidifaciens*	Heart	Attenuated	N/A	High fiber diet heart failure mice	Marques et al., 2017
**Gut bacteria structures**					
LPS	Intestine	Facilitated	LPS–TLR4–Myd88 dependent signal/NF-κB↑/Smad7↓/TGF-β1↑	Intestinal fibroblasts	Burke et al., 2010
Flagellin	Intestine	Facilitated	Flagellin-TLR Myd88 dependent signal/phosphorylated eIF2α↓, phosphorylated 4EBP1↑/ECM protein translation↑	Intestinal myofibroblast	Zhao et al., 2020
LPS	Intestine	Facilitated	N/A	Fibrocytes	Sazuka et al., 2014
PG–PS	Intestine	Facilitated	N/A	Intestinal myofibroblasts	van Tol et al., 1999
**Gut microbial metabolites**					
TMAO	Heart	Facilitated	TMAO/TGF-βR1↑, Smad2↑/cardiac fibroblast differentiation↑	High choline diet mice, TMAO contained diet mice, cardiac fibroblast	Yang et al., 2019
TMAO	Kidney	Facilitated	TMAO/Smad3↑/TGF-β signaling↑	High-fat diet mice, TMAO-supplement diet mice	El-Deeb et al., 2019
PCS	Kidney	Facilitated	Intracellular accumulation↑/activated NADPH oxidase↑/TGF-β signaling↑	HK-2 cells, 5/6-nephrectomy mice	Tang et al., 2015

*LPS, lipopolysaccharide; TLR, toll like receptor; Myd88, myeloid differentiation primary response gene 88; NF-κB, nuclear factor-κB; Smd7, small mothers against decapentaplegic protein 7; TGF-β1, tissue growth factor-β1; PG–PS, polymers peptidoglycan–polysaccharide; eIF2α, eukaryotic initiation factor 2α; 4EBP1, eukaryotic translation initiation factor 4E-binding protein 1; ECM, extracellular matrix; N/A, not applicable; TL1A, tumor necrosis factor-like cytokine 1A; AIEC, adherent/invasive *E. coli*; Ybt, yersiniabactin; IL, interleukin; ST2, growth stimulation expressed gene 2; DSS, dextran sodium sulfate; ASF, altered Schaedler flora; MAT, mesenteric adipose tissue; TMAO, trimethylamine N-oxide; TGF-βR, tissue growth factor-β receptor; PCS, *p*-cresyl sulfate.*

**FIGURE 2:**
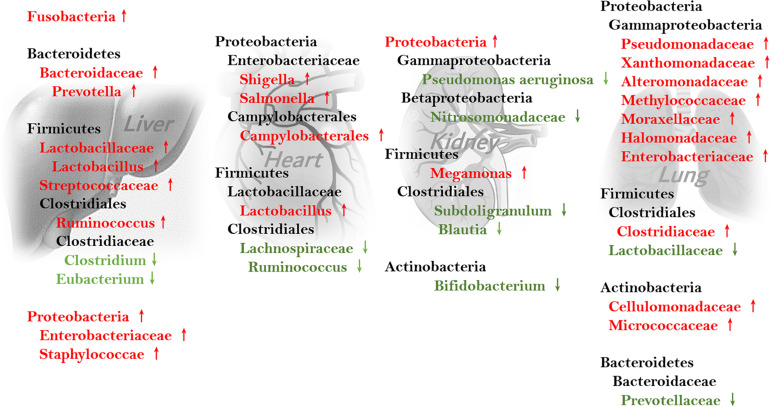
Feces-associated microbiota alterations involved in fibrosis of different organs.

Intestinal microbiome composition changes during fibrosis are well-studied in hepatic, cardiac, and pulmonary fibrosis, and are different and even at times paradoxical between organs (see Figure 2). For example, the genus *Ruminococcus*, which facilitates intestinal fibrosis and is increased in cirrhosis, is decreased in patients with cardiac insufficiency. Variations in intestinal *Firmicutes* species are also heterogenous in different fibrotic organs. Conversely, some bacteria are similarly increased in different organs. *Streptococcus* is thought to induce intestinal fibrosis and is enriched in cirrhosis. *Lactobacillus* is abundant in patients with hepatic and cardiac fibrosis. Further, *Proteobacteria* overgrowth is found in patients with cirrhosis and cardiac insufficiency.

Based on this aforementioned comprehensive analysis, clues from other organs can be gained to further study the issue of gut microbiota and intestinal fibrosis. Certain microbes and microbial metabolites appear to be positively related to fibrosis regarding to other organs, and these have never been previously studied in the intestine. Whether they induce intestinal fibrosis and their underlying mechanism is worthy of further study.

On the other hand, different microbial changes have been identified in patients with cirrhosis with different fibrotic etiologies and cirrhotic disease periods. By analyzing variations in specific intestinal microbes, researchers have constructed models to predict the development of cirrhosis and its complications (Bajaj et al., 2014; Caussy et al., 2019). Recently, a similar microbial prediction model was generated for pulmonary fibrosis (Gong et al., 2021). Therefore, examining the dysbiosis that occurs in intestinal fibrosis and its value in disease prediction may also be worthwhile.

Finally, almost all research has involved bacteria and there are currently limited studies on the impacts of non-bacterial microbes on fibrosis. An examination of these will also expand our understanding of the relationship between gut microbiota and intestinal fibrosis.

## Author Contributions

XZ and ZZ designed the study. SZ, NL, CL, and RM wrote the manuscript. CL collected the data. DW and TL analyzed the data. MC and ZZ revised the manuscript. All authors approved the final version.

## Conflict of Interest

The authors declare that the research was conducted in the absence of any commercial or financial relationships that could be construed as a potential conflict of interest.
